# The IAP Antagonist SM-164 Eliminates Triple-Negative Breast Cancer Metastasis to Bone and Lung in Mice

**DOI:** 10.1038/s41598-020-64018-z

**Published:** 2020-04-24

**Authors:** Wei Lei, Rong Duan, Jinbo Li, Xin Liu, Alissa Huston, Brendan F. Boyce, Zhenqiang Yao

**Affiliations:** 1 Department of Pathology and Laboratory Medicine, and Center for Musculoskeletal Research, University of Rochester Medical Center, Rochester, NY 14642 USA; 2 Department of Medical Imaging, Henan University First Affiliated Hospital, 357 Ximen Street, Kaifeng, Henan 475001 P.R. China; 3 Department of Medicine, Hematology/Oncology, University of Rochester Medical Center, Rochester, NY 14642 USA

**Keywords:** Drug development, Preclinical research, Breast cancer

## Abstract

The most challenging issue for breast cancer (BC) patients is metastasis to other organs because current therapies do not prevent or eliminate metastatic BC. Here, we show that SM-164, a small molecule inhibitor, which degrades inhibitor of apoptosis proteins (IAPs), eliminated early-stage metastases and reduced progression of advanced BC metastasis from MDA-MB-231 BC cells in bones and lungs of nude mice. Mechanistically, SM-164-induced BC cell death is TNFα-dependent, with TNFα produced by IL-4-polarized macrophages triggering MDA-MB-231 cell apoptosis in combination with SM-164. SM-164 also inhibited expression of RANKL, which mediates interactions between metastatic BC and host microenvironment cells and induces osteoclast-mediated osteolysis. SM-164 did not kill adriamycin-resistant BC cells, while adriamycin inhibited SM-164-resistant BC cell growth, similar to parental cells. We conclude that SM-164 is a promising therapeutic agent for early stage bone and lung metastasis from triple-negative breast cancer that should be given prior to conventional chemotherapy.

## Introduction

Breast cancer (BC) accounts for nearly a quarter of all cancers in women worldwide. It is estimated that 268,600 women will be diagnosed with invasive BC in the US in 2019 and 41,760 will die from it^1^. The most challenging issue for patients with BC is metastasis to other organs, including bone, lung, liver and brain; this causes about 90% of BC deaths. For decades, BC treatment has included surgery, radiation therapy (RT), chemotherapy (CT), and/or hormonal therapy. However, none of the current therapies effectively prevents or eliminates BC. Adjuvant CT benefits only a small proportion (5-10%) of patients^2^. Similarly, RT has resulted in only a 5% reduction in the 15-year BC mortality rate^3^. One reason for the poor therapeutic outcome could be that many patients already have micro-metastases when their primary cancers are diagnosed^4–6^. Bone metastases are distinct from metastases to other organs because of cancer-associated osteolysis due to enhanced osteoclast (OC) formation and activity and associated release of cancer-promoting proteins from the resorbed bone matrix, including TGFβ^7,8^. Current standard anti-resorptive drugs (bisphosphonates and the RANKL inhibitor, denosumab) inhibit bone resorption and reduce skeletal-related events (SREs)^9,10^, but they do not prolong patient survival, and 30-50% of BC patients on these drugs still develop new bone metastases^9,11,12^.

The development and progression of BC metastases depend on interactions between the cancer cells and the host organ microenvironment. For example, circulating cancer cells can be attracted to bone by osteoblasts (OBs) or their progenitors, mesenchymal stem cells (MSCs), by expression of proteins, such as integrins, chemokines, Notch, nestin, and osteopontin by these cells^12^, and also via interactions between RANKL on OBs/MSCs and RANK expressed by cancer cells^13,14^. Cancer cells in turn promote OB/MSC production of more RANKL to enhance OC formation, causing osteolysis^11,15,16^ and release of factors from bone matrix, including TGFβ, which can inhibit OB formation^17^ and OB-mediated repair of lytic lesions^7,8^. In addition, MSCs in lung secrete CCL5 to recruit CD4^+^FOXP3^+^ Treg cells, which produce RANKL and this promotes seeding of BC cells in lungs^18^.

In addition to directly inhibiting OC formation by negatively regulating NF-κB signaling in OC precursors through degradation of NF-κB-inducing kinase (NIK)^19^, TNF receptor associated factor 3 (TRAF3), an adaptor protein that interacts with cytokine receptors, also maintains MSC differentiation into OBs and inhibits their expression of RANKL, as we reported recently^17^. cIAP1 and cIAP2 cooperate with TRAF2 to degrade TRAF3 in a variety of cell types, resulting in NF-κB activation^20–22^. TGFβ1 induces ubiquitin-mediated degradation of TRAF3 in MSCs to inhibit OB differentiation directly, and this degradation also results in increased RANKL production by MSCs, leading to enhanced osteolysis^17^.

Triple-negative breast cancer, which lacks expression of estrogen receptor (ER), progesterone receptor (PR) and human epidermal growth factor receptor 2 (Her-2), is the most aggressive subtype of breast cancer and has a poor prognosis. Targeted therapies against ER, PR and Her-2 are ineffective for patients with triple-negative BC. Here, we report that the bivalent second mitochondria-derived activator of caspases (Smac) mimetic, SM-164^23,24^, which was developed to kill cancer cells by degrading both cIAP (cellular inhibitor of apoptosis) and XIAP (X-linked inhibitor of apoptosis) proteins^23,24^, eliminates early stage metastatic BC and significantly reduces the progression of advanced bone and lung metastases from the triple-negative human breast cancer cell line, MDA-MB-231, in a mouse model.

## Results

### SM-164 prevents the establishment and progression of early-stage metastasis of breast cancer cells in bone and lungs

**Figure 1:**
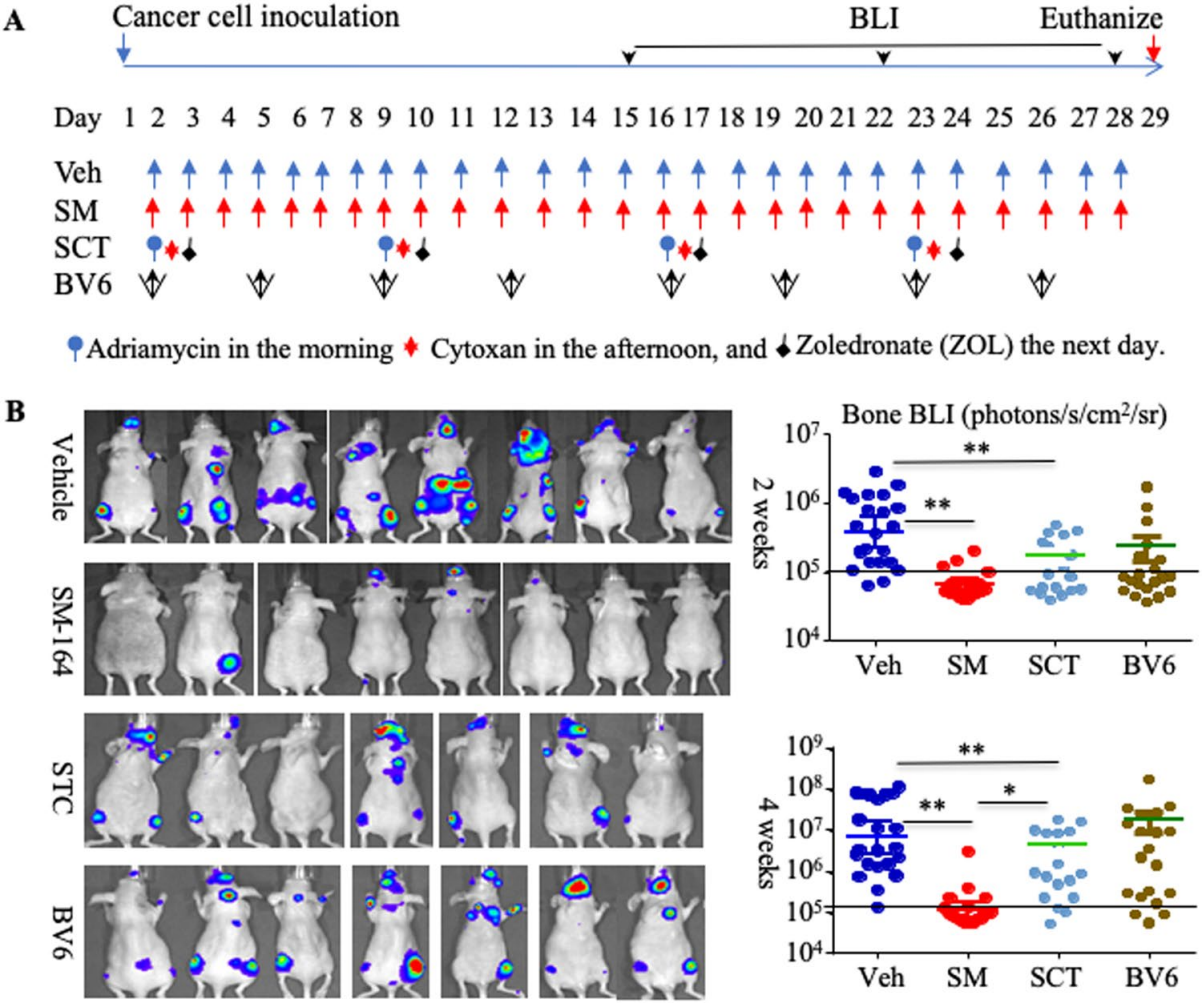
SM-164 inhibits the growth of metastatic MDA-MB-231 cancer cells in bone. (**A**) Scheme showing that MDA-MB-231^luci^ cells were inoculated into the left cardiac ventricle of 7-wk-old female athymic nude mice followed by treatment with vehicle, SM-164, standard chemotherapy (SCT) or BV6, starting on the 2^nd^ day. (**B**) Mice were monitored weekly by BLI. Representative BLI images and BLI signal intensity in hind limbs after 2 and 4 weeks of treatment are shown, 7-8 mice each group, in which BLI signal intensity of each leg was calculated individually. *p < 0.05 and **p < 0.01. One-way ANOVA +/Dunnett test. The horizontal line at 10^5^ indicates the mean value in the mice at baseline.

To examine the effects of SM-164 on the establishment and progression of early-stage metastasis of breast cancer cells, we inoculated luciferase-expressing human MDA-MB-231^luci^ cells into the left cardiac ventricle of 7-wk-old female athymic nude mice. From the 2^nd^ day, by which time the circulating cells are likely to have colonized bone and other tissues^25^, the mice were treated with 1) vehicle, 2) SM-164, 3) combined standard chemotherapy (SCT), or 4) twice weekly injections of BV6, as a negative control, which has been reported to enhance BC bone metastasis in mice by secondarily stimulating bone resorption^26^, as illustrated in Fig. 1A. The mice were monitored weekly using bioluminescence imaging (BLI). After 2 weeks of treatment, BLI signals in hind limbs of the mice given SM-164 were significantly lower than in those given vehicle (p < 0.01, Fig. 1B). Mice given SCT also had significantly lower BLI signals in their hind limbs than vehicle-treated mice (p < 0.05, Fig. 1B), but SCT was less effective than SM-164 (p < 0.01, Fig. 1B). Similarly, after 4 weeks of treatment, both SM-164- and SCT-treated mice had significantly lower BLI signal intensity in hind limbs than vehicle-treated mice, but SM-164 was more effective than SCT (p < 0.01, Fig. 1B).

**Figure 2:**
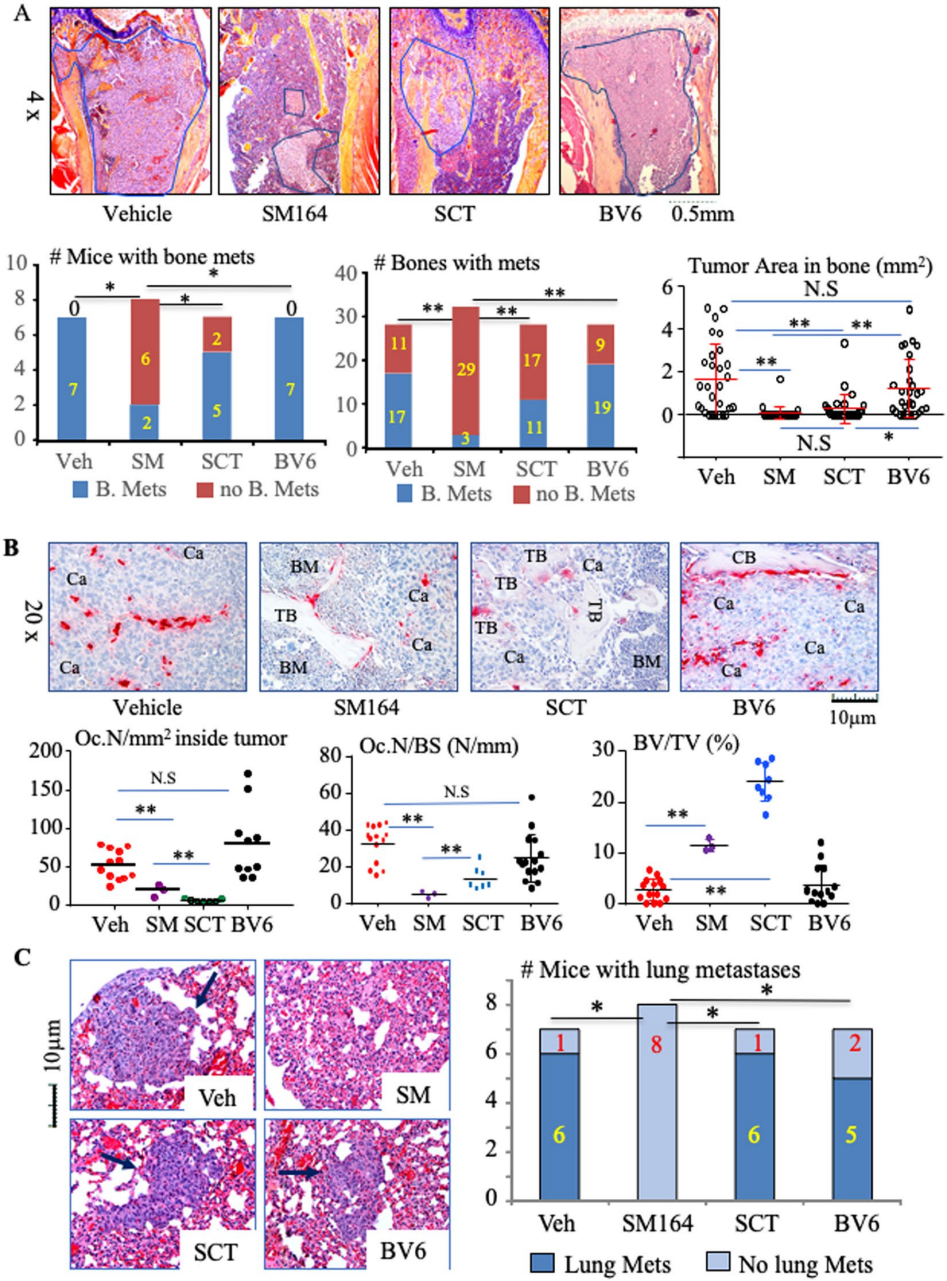
SM-164 prevents the establishment and progression of early-stage metastasis of breast cancer cells in bone and lungs. Mice, as in Fig. 1, were euthanized on day 29. (**A**) Representative H&E-stained sections of upper tibiae (upper panel) with tumor metastases outlined in blue. The frequency of bone metastasis, based on the number of mice (left of lower panel) and number of tibiae and femora (middle of lower panel) with bone metastases (mets), *p < 0.05 and **p < 0.01, non-parameter analysis. Tumor burden in bone, evaluated as tumor area in each long bone of the legs. *p < 0.05 and **p < 0.01, one-way ANOVA +/Dunnett test. (**B**) Representative TRAP**-**stained sections of tibiae (upper panel) to illustrate OCs (red staining) in metastatic tumor deposits. OC numbers inside metastatic cancer (left lower panel) and on trabecular bone surfaces (middle lower panel) as well as volume of non-resorbed trabecular bone (right lower panel). Ca = cancer, BM = normal bone marrow, CB = cortical bone, TB = trabecular bone. *p < 0.05 and **p < 0.01, one-way ANOVA +/Dunnett test. (**C**) Representative H&E-stained sections of lungs, with lung metastases arrowed, and analysis of the numbers of mice with lung metastases. *p < 0.05, non-parametric analysis.

The mice were euthanized on day 29 to evaluate metastases in bones and lungs histologically. Significantly fewer mice treated with SM-164 alone had bone metastases (2/8) compared with vehicle (7/7; p < 0.01, Fig. 2A). SM-164 also markedly reduced the total number of tibiae and femora with metastatic cancer compared with vehicle, 3/32 bones (9%) versus 20/28 (71%), p < 0.01 (Fig. 2A). Tumor area was also significantly lower in bones of the mice treated with SM-164 than in mice treated with vehicle (Fig. 2A, 0.3 ± 0.7 versus 6.5 ± 3.8 mm^2^, p < 0.01). Although SCT did not reduce the numbers of mice or the numbers of bones with metastases, it significantly reduced the tumor area in bone compared with vehicle (Fig. 2A, 1.2 ± 1.6 mm^2^, p < 0.01 vs. vehicle).

OCs formed among the metastatic tumor cells distant from bone surfaces within tumor deposits inside affected bones, and both SM-164 and SCT significantly reduced the numbers of OCs inside the tumor area and on the remaining bone surfaces (Fig. 2B). Non-functional, “giant osteoclasts” similar to those described in samples of bone from humans treated with bisphosphonates (BPs)^27^ were present on the trabecular surfaces of mice treated with SCT. These OCs also had condensed nuclei and cytoplasmic contraction, features typical of apoptosis, which the ZOL in SCT and other BPs induce as part of their inhibitory effects on osteoclasts^28^. Of note, none of the three long bones in the two SM-164-treated mice with metastases had cortical erosion induced by the metastatic cancer cells in comparison with extensive trabecular and cortical destruction in vehicle-treated mice (Fig. 2A,B). Consistent with these findings, the trabecular bone volume in the long bones of SM-164-treated mice with metastases was significantly higher than that in mice treated with vehicle, but was significantly lower than that in mice treated with SCT (Fig. 2B, lower right panel), reflecting the well-known effects of nitrogen-containing BPs to increase bone mass in growing mice by inhibiting resorption of newly-formed bone in metaphyses^28^. In addition, unlike the typical effects of ZOL in SCT, SM-164 alone did not increase the trabecular bone volume or affect OC numbers in vertebrae that did not have metastases (Supplementary Fig. 1).

Importantly, SM-164 also prevented the development of lung metastases. None of the 8 mice treated with SM-164 had metastases in histologic sections of their lungs compared with 6/7 of vehicle- and SCT-treated mice (Fig. 2C). We also examined livers and brains, two other common sites for breast cancer metastasis, but did not find metastases in them in any group, probably because this MDA-MB-231 cell line preferentially metastasizes to bone and lung^29,30^.

Unlike the findings in a previous study in which mice given twice weekly doses of BV6 developed enhanced bone metastasis^26^, we found that BV6 did not promote or inhibit the development of BC bone metastasis, as assessed by BLI signal intensity in their hind limbs (Fig. 1B), or numbers of bones with metastases, tumor burden in bone or the numbers of OCs associated with metastases, as assessed histologically (Fig. 2A,B). In addition, BV6 treatment did not reduce metastases to lungs (Fig. 2C).

### SM-164 inhibits the progression of advanced breast cancer metastases in bone and lungs

**Figure 3:**
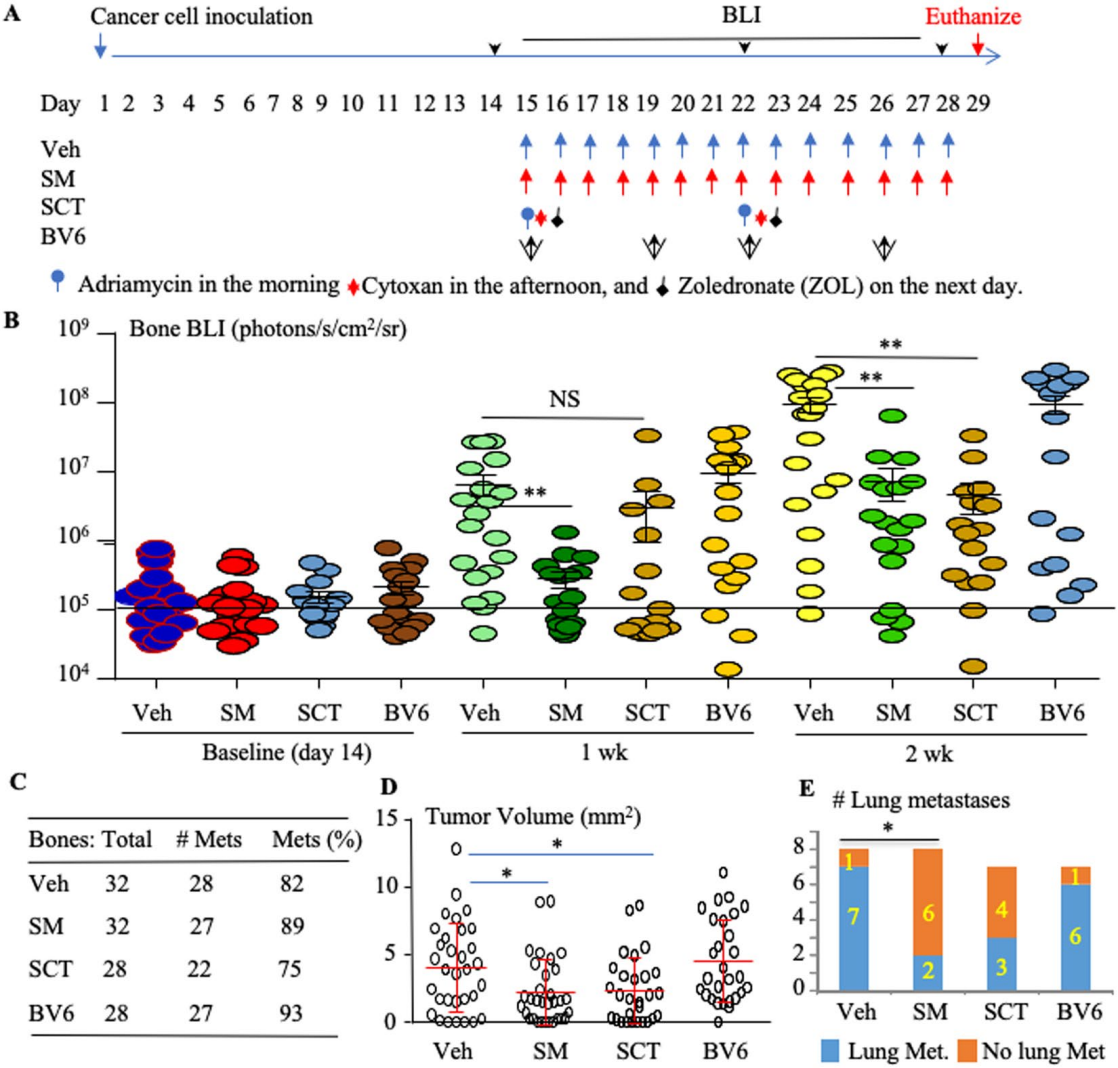
SM-164 inhibits the progression of advanced breast cancer metastases in bone and lungs. MDA-MB-231^luci^ cells were inoculated into the left cardiac ventricle of 7-wk-old female athymic nude mice. After 2 weeks, mice with established bone metastases, determined by BLI, were randomly divided into 4 groups, 7-8 mice per group, which were treated with vehicle (Veh), SM-164 (SM), SCT or BV6, as illustrated in (**A**). Tumor growth in the legs was evaluated by BLI signal intensity, calculated on individual legs, after 1 and 2 weeks of treatment (**B**). *p < 0.05 and **p < 0.01, one-way ANOVA +/Dunnett test. The horizontal line at 10^5^ indicates the mean value in the mice at baseline. Mice were euthanized on day 29; (**C**) the total numbers and percentage of tibiae and femora (Bones) with metastases (Mets), *p < 0.05, non-parameter analysis; (**D**) tumor volume were determined on H&E-stained sections, as in Fig. 2A; *p < 0.05, one-way ANOVA +/Dunnett test. (**E**) The numbers of mice with lung metastases was evaluated (*p < 0.05, non-parametric analysis), as in Fig. 2C.

We also evaluated the effects of SM-164 on the progression of advanced BC metastases, as illustrated in Fig. 3A. 14 days after intra-cardiac inoculation of MDA-MB-231^luc^ BC cells, mice were examined with BLI to confirm that bone metastases had been established (Fig. 3B). Mice with metastases were then allocated to the groups illustrated in Fig. 3A, and treatment was started on day 15. BLI signal intensity was significantly reduced in the long bones of mice treated with SM-164 after 1 and 2 weeks and in mice given SCT after 2 weeks compared with vehicle (Fig. 3B, p < 0.01). Although neither SM-164 nor SCT eliminated the metastases from bone (Fig. 3C), both of them significantly reduced the area of established metastases in bone evaluated by histology compared with vehicle (Fig. 3D). Importantly, SM-164 alone also reduced the number of lungs with metastases compared with vehicle when the treatment was begun after 2 weeks (Fig. 3E). In contrast, BV6 did not accelerate or reduce the progression of established bone or lung metastases evaluated by BLI and histology compared with vehicle (Fig. 3A–E).

### SM-164 induces apoptosis of breast cancer cells in combination with TNFα released by tumor-associated macrophages

**Figure 4:**
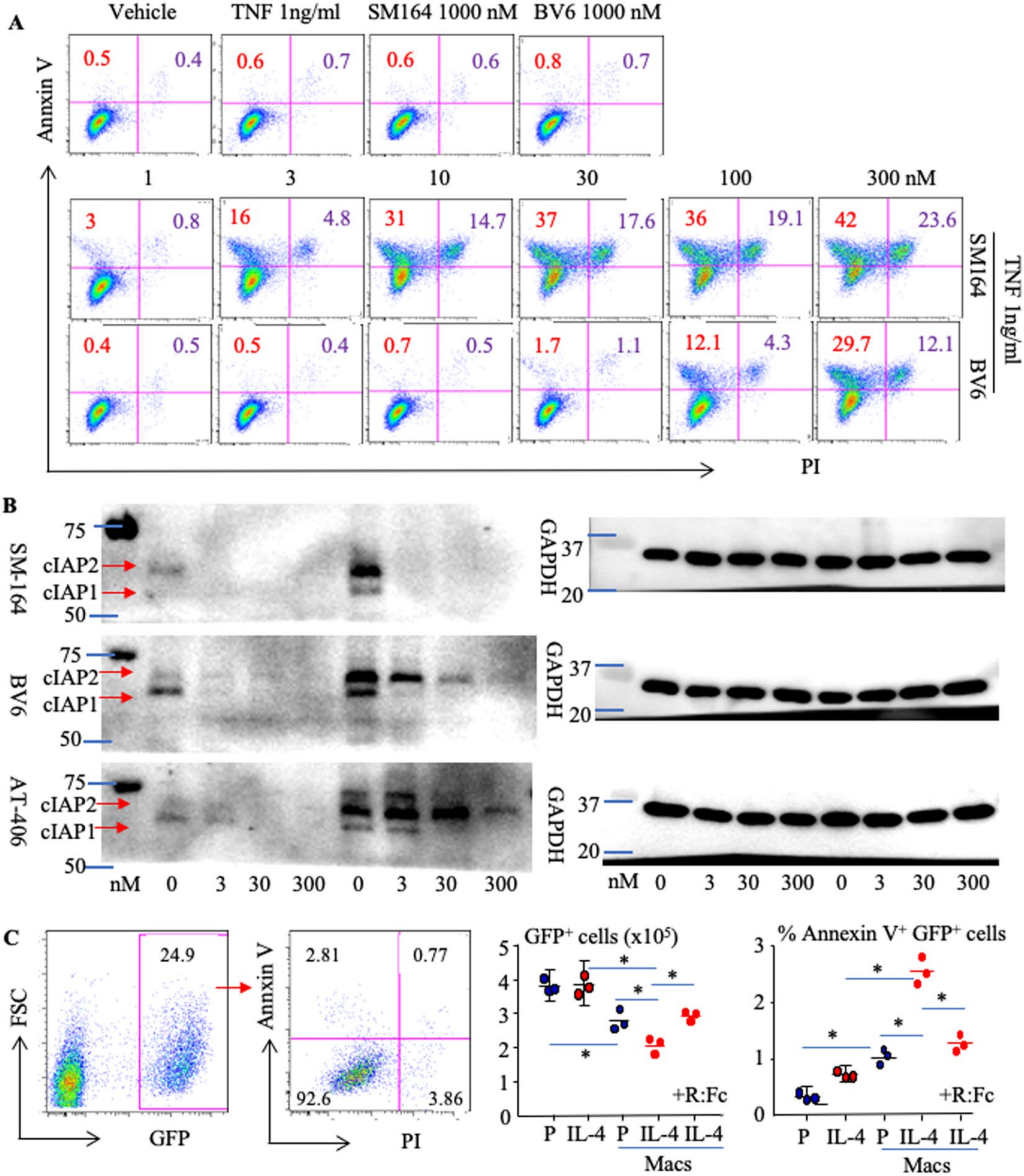
SM-164 induces apoptosis of breast cancer cells in combination with TNFα released by tumor-associated macrophages. (**A**) The parental MDA-MB-231 cells were treated with vehicle, TNFα (1 ng/ml) or TNFα + the indicated doses of SM-164 or BV6 overnight. Annexin V^+^PI^+/−^ apoptotic cells were analyzed by flow cytometry. (**B**) Parental MDA-MB-231 cells were treated with the indicated doses of SM-164, BV6 or AT-406 alone, or with a combination of them plus TNFα (1 ng/ml) for 8 hours. Cell lysates were used to test protein levels of cIAP1/cIAP2 and GAPDH. (**C**) 1 × 10^4^ GFP^+^ MDA-MB-231 cells were cultured alone or together with WT mouse BM cells in the presence of M-CSF +/− PBS (P) or IL-4 for 3 d, during which 3 nM SM-164 and 1 μg/ml TNFR:Fc (R:Fc) were added for the last 16 hr (last group on right +R:Fc). Annexin V^+^PI^+/−^ apoptotic cells in the GFP^+^ population were analyzed by flow cytometry (left panel). The total number of GFP^+^ cells (left graph) was calculated based on % of GFP^+^ cells in the total cell number, and % of Annexin V^+^PI^+/−^ apoptotic cells in the GFP^+^ population (right graph) was calculated. *p < 0.05 and **p < 0.01, one-way ANOVA +/Dunnett test.

SM-164 was previously developed to kill cancer cells by degrading both cIAP and XIAP^23,24^. We found that neither IAP antagonist (SM-164 and BV6) alone (up to 1000 nM) induced MDA-MB-231 cell apoptosis *in vitro* (Fig. 4A, upper panel), consistent with published reports that low doses of SM-64^24,31^ or BV6^32^ have little effect on cancer cell apoptosis or viability. Similarly, TNFα alone did not induce BC death (Fig. 4A). In contrast, low doses of TNFα (1 ng/ml) and SM-164 (3 nM) given in combination markedly induced MDA-MB-231 cell apoptosis (Fig. 4A, middle panel), consistent with the findings that induction of cancer cell apoptosis by IAP antagonists largely depends on the presence of TNFα^24,33^. Of note, the potential of SM-164 to induce MDA-MB-231 cell apoptosis in the presence of TNFα was 30-fold higher than that of BV6, starting around 1 nM vs. 30 nM, respectively (Fig. 4A, middle and lower panel). Similarly, a combination of low doses of SM-164 and TNFα induced apoptosis of ER^+^ human MCF-7 BC cells (Supplementary Fig. 2).

We found that among the IAP antagonists we tested, including SM-164, BV6 and AT-406^34^, SM-164 most effectively degraded cIAP1 and cIAP2 in MDA-MB-231 cells (Fig. 4B). Of note, a low dose of TNFα (1 ng/ml) markedly increased cIAP1 and cIAP2 protein levels (Fig. 4B). In addition, a low dose of SM-164 (3 nM) completely degraded cIAP1 and cIAP2, while cells treated with 300 nM of BV6 or AT-406 still had low levels of cIAP1 and cIAP2. These findings suggest that SM-164 has at least 100-fold greater efficacy than BV6 or AT-406 to degrade cIAP1 and cIAP2, paralleling its greater potency to kill cancer cells in the presence of TNFα (Fig. 4B).

Macrophages are one of the main sources of TNFα and are among the most abundant non-neoplastic cells in the tumor microenvironment^35^. Macrophages are classified as inflammatory (M1) and anti-inflammatory (M2), which are linked to Th1- and Th2-type immune responses, respectively^36^. Tumor-associated macrophages (TAMs) exhibit mainly a M2 phenotype^35^. IL-4 polarizes macrophages to a M2 phenotype^35,37^. Thus, we evaluated if IL-4 stimulates TNFα production by macrophages to trigger SM-164-induced BC apoptosis. We found that IL-4 + SM-164 did not trigger MDA-MB-231 apoptosis (Fig. 4C). However, IL-4-polarized macrophages from WT mice in combination with SM-164, slightly but significantly increased apoptosis of the cancer cells and decreased the total number of GFP^+^ cells (Fig. 4C). Importantly, addition of a TNFα receptor/IgG:Fc fusion protein (TNFR:Fc)^38,39^ blocked apoptosis induced by IL-4-polarized macrophages and SM-164 (Fig. 4C), suggesting that IL-4-polarized WT macrophages produce TNFα to trigger SM-164 induction of BC apoptosis.

### SM-164 promotes osteoblast and inhibits osteoclast formation and reduces RANKL^+^ cells in bone and lung by preventing degradation of TRAF3

**Figure 5:**
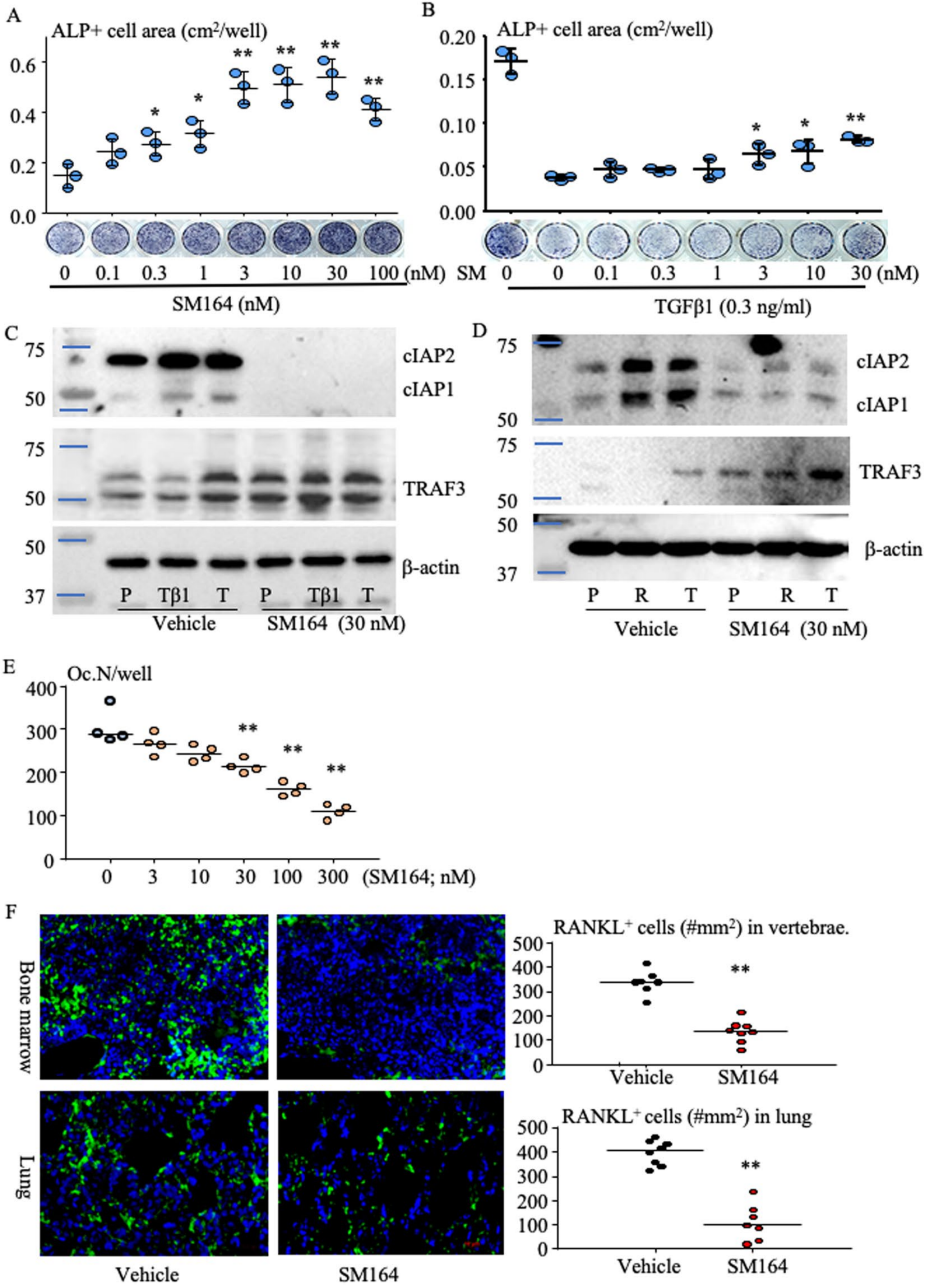
SM-164 promotes osteoblast and inhibits osteoclast formation and reduces RANKL^+^ cells in bone and lung by preventing degradation of TRAF3. (**A**) Bone-derived mesenchymal progenitor cells (BdMPCs) from C57Bl6 mice were treated with 25 μg/ml L-ascorbic acid and 5 mM β-glycerophosphate to induce OB differentiation in the presence of the indicated doses of SM-164 for 7 d. ALP staining was performed to evaluate ALP^+^ OB area. *p < 0.05 and **p < 0.01 vs. vehicle. (**B**) Effects of SM-164 on ALP^+^ OB differentiation in the presence of TGFβ1, evaluated as in (A). *p < 0.05 and **p < 0.01 vs. TGFβ1 alone; one-way ANOVA +/Dunnett test. (**C**) BdMPCs were treated with TGFβ1 (Tβ1), 1 ng/ml, or TNFα (T), 3 ng/ml, +/− SM-164. TRAF3, cIAP1/2 and β-actin protein levels were assessed in cell lysates by Western blot (WB). Each gel image was cropped from same gel, and full-length gel images are presented in Supplementary Fig. 5A. (**D**) WT mouse BM cells were cultured with M-CSF for 2 d to generate macrophages, which were then treated with PBS (P), RANKL (R) or TNFα (T) in the presence of vehicle or SM-164 for 8 hr. cIAP1/2 and TRAF3 protein levels were assessed by WB. Each gel image was cropped from the same gel, and full-length gel images are presented in Supplementary Fig. 5B. (**E**) WT mouse macrophages, generated as in (**D**), were treated with 10 ng/ml RANKL in the presence of the indicated doses of SM-164 for an additional 2 d. TRAP staining was performed to evaluate OC numbers. **p < 0.01 vs. culture without SM-164, one-way ANOVA +/Dunnett test. (**F**) Sections from paraffin-processed vertebral bones without metastasis (upper panel) and lungs (lower panel) from vehicle- and SM-164-treated mice, as in Figs. 1 and 2, immunostained for RANKL (green) with DAPI counterstaining (blue). RANKL^+^ cells were counted on captured fluorescent images (40X). **p < 0.01 vs. vehicle, unpaired Student *t* test. All *in vitro* experiments were repeated 3 times with similar results.

Interestingly, SM-164 dose-dependently (from 0.3 nM) increased ALP^+^ OB differentiation (Fig. 5A) from human MSCs, even in the presence of TGFβ1 (Fig. 5B), which is known to inhibit OB differentiation^8^, although it only partly prevented TGFβ1-induced inhibition of OB differentiation (Fig. 5B). Importantly, SM-164 blocked TGFβ1-induced TRAF3 degradation in MSCs associated with reduction of cIAP1 and cIAP2 proteins (Fig. 5C). SM-164 also inhibited TRAF3 degradation, associated with reduced cIAP1 and cIAP2 proteins, and dose-dependently (10–300 nM) inhibited RANKL-induced OC formation (Fig. 5D,E). SM-164 had a stronger effect than BV6 to inhibit OC, stimulate OB differentiation and prevent TRAF3 degradation (Supplementary Fig. 3). Consistent with the above findings, there were significantly fewer RANKL^+^ cells in sections of vertebral bone without metastasis and lungs from SM-164-treated mice than in sections from vehicle-treated mice, as assessed by immunofluorescence (Fig. 5F). This is consistent with our recent report that TGFβ1 degrades TRAF3 in MSCs to increase RANKL production through activation of NF-κB^17^ and that expression of RANKL by MSCs (OBs/fibroblasts) mediates the interactions of circulating cancer cells with the host microenvironment not only in bone^13,14^, but also in lung^18,40^ to promote cancer metastasis.

### ADR-resistant (R) MDA-MB-231 cells are also resistant to SM-164, while SM-164-R MDA-MB-231 cells are sensitive to ADR

**Figure 6:**
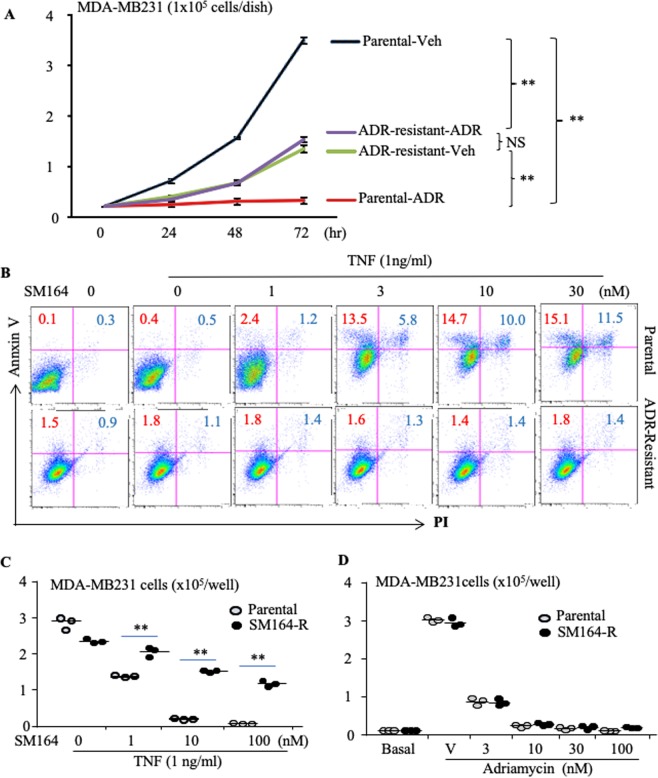
ADR-resistant (R) MDA-MB-231 cells are also resistant to SM-164, while SM-164-R MDA-MB-231 cells are sensitive to ADR. (**A**) Growth curve of the parental and ADR-R MDA-MB-231 cells, starting at 1 × 10^4^ cells/well, in a well of 6-well-plates treated with 30 ng/ml ADR or vehicle, and passaged to 60- and 100-mm dishes, respectively, when untreated cells grew to sub-confluence. 3 wells/group, **p < 0.01, one-way ANOVA +/Dunnett test. (**B**) Parental and ADR-R MDA-MB-231cells were treated with the indicated doses of SM-164 plus 1 ng/ml of TNFα overnight. Annexin V^+^PI^+/−^ apoptotic cells were analyzed by flow cytometry. (**C**) 0.5 × 10^4^ parental and SM-R MDA-MB-231 cells in 60 mm dishes were cultured overnight followed by treatment with 1 ng/ml TNFα plus the indicated doses of SM-164 for 3 days, and surviving cells were counted. (**D**) The parental and SM-164-R cells (0.5 × 10^4^ cells in 60 mm dishes) were treated with the indicated doses of ADR for 6 days and cell numbers were counted after digestion with 0.2% trypsin. **p < 0.01 vs. the respective parental cells, one-way ANOVA +/Dunnett test. The experiments were repeated 3 times with similar results.

Several IAP antagonists, including GDC-0917/CUDC-427, LCL161 and AT-406/Debio1143, and the bivalent agent, TL32711/birinapant, have been tested in clinical trials for the treatment of cancers, including breast, lung, ovarian and colon carcinomas, melanoma, lymphoma, and leukemia^41–46^. Some patients benefitted from IAP antagonist therapy, but many others did not, probably because most of the recruited patients in these trials had advanced-stage cancers^41–46^. It is likely that these patients had already received standard chemotherapy. We speculate that the patients who did not benefit from IAP antagonists might already have developed resistance to them due to multidrug resistance resulting from previous conventional chemotherapy, such as ADR^47^. To test this hypothesis, we developed ADR-resistant MDA-MB-231 cells by treating the cells long-term with low doses of ADR (Fig. 6A). We found that SM-164, up to 30 nM, in combination with TNFα, did not induce apoptosis of ADR-resistant MDA-MB-231 cells (Fig. 6B). We then developed SM-164-resistant MDA-MB-231 cells (Fig. 6C) to test if current standard chemotherapy could kill these cells. Interestingly, ADR inhibited the growth of SM-164-resistant MDA-MB-231 cells similar to the parental cells (Fig. 6D).

## Discussion

This the first report that an IAP antagonist (SM-164) administered alone effectively eliminates and prevents the progression of early stage metastasis of human MDA-MB-231 cells to bone and lungs *in vivo* in a standard mouse metastasis model (Figs. 1 and 2). In contrast, current combined SCT, including ADR, CYT and ZOL, did not eliminate bone metastases, although it significantly inhibited tumor growth and metastatic cancer-associated bone destruction (Figs. 1 and 2). Adjuvant chemotherapy is widely used after surgical removal of primary breast cancers to kill cancer cells in the circulation and in micro-metastases in organs to prevent recurrence and metastasis, but it benefits only a small proportion (5-10%) of patients and has significant side-effects^2^. We did not observe any serious side-effects in mice treated for up to 4 weeks with SM-164 and they had normal body weight at the end of the experiment (Supplementary Fig. 4). Thus, our findings suggest that SM-164 could be given to patients before or after surgical removal of their primary breast cancers to kill off cancer cells at sites of early metastasis.

We chose to initiate therapy on the 2^nd^ day after BC cell inoculation into the left cardiac ventricle of mice because the cancer cells, like injected myeloma cells^25^, will already have colonized niches adjacent to bone surfaces and in other organs, and some of them will still be in the circulation, which is similar to early stage micro-metastasis in humans. Interestingly, like current combined SCT, SM-164 reduced the progression of advanced BC metastases in bone, evaluated by dynamic changes in BLI signal intensity and tumor volume evaluated histologically in long bones (Fig. 3). Importantly, SM-164, but not SCT, significantly reduced advanced stage lung metastasis (Fig. 3E), supporting our suggestion that this IAP antagonist could potentially be very useful as an adjuvant therapy after surgical removal of primary cancers to effectively eliminate remaining micro-metastases.

One explanation for SM-164 effectively eliminating early stage metastases and reducing the progression of advanced stage BC metastasis to bone and lung in our study is that it kills BC cells in combination with TNFα produced locally by TAMs. However, macrophages, even those polarized by IL-4, triggered BC cell apoptosis less effectively in the presence of SM-164 than direct treatment with TNFα (Figs. 4, 6 & Supplementary Fig. 2). In particular, SM-164 also induces the death of macrophages^48^. These findings could explain our observations that SM-164 alone only slightly reduced the progression of advanced metastatic BC, but did not eliminate it, similar to combined SCT (Fig. 3). T cells are another source of TNFα^49^, which could explain why CAT-T cells synergize with an IAP antagonist to treat cancer^50^. In particular, IAP antagonists can augment human and mouse T cell responses and cytokine production to physiologically relevant stimuli^51,52^. Thus, T cell deficiency in nude mice could be another reason why SM-164 alone had limited efficacy in mice with advanced BC metastases. However, it is also possible that the dose of SM-164 that we administrated was insufficient. Together, our findings suggest that a combination of SM-164 and TNFα could be an effective therapy for advanced BC metastases. However, TNFα can result in serious side effects, including systemic shock and widespread inflammatory responses, due to its cytotoxic, cytostatic, and immunomodulatory properties^53–55^. Thus, further studies are required to determine the downstream signaling from TNFα that synergizes with SM-164 or other IAP antagonists to induce cancer cell apoptosis in order to design a novel effective combined therapeutic approach.

Another explanation for SM-164 effectively eliminating early BC metastasis is that it blocks interactions of cancer cells with the host microenvironment and indirectly reduces BC-enhanced OC formation by inhibiting RANKL expression by MSCs/OBs in the host organs, including bone marrow and lungs through stabilization of TRAF3 (Fig. 5). This is supported by our findings that: 1) SM-164 reduced OC formation in long bones with metastatic BC (Fig. 2B), but not in vertebral bones without metastases (Supplementary Fig. 1); 2) the dose of SM-164 to prevent TGFβ1-induced TRAF3 degradation and to stimulate OB differentiation *in vitro* is low and similar to the dose that kills breast cancer cells (~1 nM) (Figs. 4A and 5A–C); 3) in contrast, much higher doses of SM-164 (~30 nM) are needed to directly inhibit OC formation *in vitro* (Fig. 5E); and importantly, 4) SM-164 significantly inhibited expression of RANKL not only in vertebrae, but also in lungs (Fig. 5F). This is consistent with reports that lung tissues express RANKL^56^ and TGFβ1 stimulates expression of CCR7 by MSCs in lungs to attract circulating BC cells and support their growth in lung^40^.

Our findings that SM-164 cannot not kill ADR-resistant cancer cells (Fig. 6B), possibly due to ADR induction of multidrug resistance (MDR)^47^, and that SM-164-resistant MDA-MB-231 cells are sensitive to ADR, similar to the parental cells (Fig. 6D), could be very helpful to guide future clinical trials. The development of MDR continues to be a major hurdle in the treatment of patients with advanced cancer. Upregulation of a wide-range of ATP-dependent efflux pumps, in particular, ABCB1 (P-glycoprotein or MDR1), is a well-recognized drug resistance mechanism^57,58^. Our RNA-seq analysis indicated that ABCB1 mRNA levels in ADR-resistant MDA-MB-231 cells were 487-fold higher than in the parental cells (data not shown). We do not expect that an inhibitor of P-glycoprotein would enhance the effect of an IAP antagonist in the treatment of ADR-resistant BC patients because P-glycoprotein inhibitors have not overcome drug resistance in patients. IAP antagonists induce apoptosis by activating caspases, which results in cytochrome-C release from mitochondria into the cytosol^59^. We found that SM-164-resistant MDA-MB-231 cells did not over-express known MDR-related genes (data not shown). This could explain why SM-164-resistant cells responded similarly to ADR as their parental cells (Fig. 6D). Therefore, we recommend that for neo-adjuvant chemotherapy, an IAP antagonist should be given before regular chemotherapy to eliminate micro-metastases and circulating cancer cells and thus prevent relapse and metastasis after surgical resection of primary tumors. Our findings also suggest that for conventional adjuvant chemotherapy, an IAP antagonist should be given to patients immediately after primary cancers are surgically excised prior to and ideally followed by current standard chemotherapy in order to completely eliminate circulating or micro-metastatic cancer cells. New clinical trials could test these possibilities, using an IAP antagonist, such as SM-164, and in particular those compounds that have been confirmed to be safe in clinical trials^41–46^.

Pharmacokinetic studies indicate that the plasma half-life of most IAP antagonists is about 4-7 hours in humans^42–44,60^. However, some of them have been administrated weekly^42,60^. Birinapant has a longer plasma half-life of 30-35 hours, but it also has been given weekly, and there was no accumulation in plasma at day 15 in a weekly dosing regimen^46^. Further studies are needed to determine if the poor response to these IAP antagonists in patients with malignancy is due to the relatively short peak concentrations that are achieved in previous trial dosing regimens. The twice weekly dosing of BV6 in our study was originally designed as a negative control since it has been shown to enhance BC bone metastasis^26^. Mice with BC metastases did not response to this BV6 regimen (Figs. 1 and 2) likely because this dosing did not effectively kill the cancer cells. Other possible reasons to explain why SM-164, but not BV6 therapy, inhibited BC metastasis *in vivo* in our study are that the concentration of SM-164 that killed MDA-MB-231 cells *in vitro* was 30-fold lower than BV6 (Fig. 4A), and SM-164 prevented TRAF3 degradation in OB and OC progenitor cells more effectively than BV6 (Supplementary Fig. 3). Thus, further studies will be required to determine if higher and/or continuous doses of IAP antagonists, in particular those that have been confirmed to be safe in clinical trials^41–46^ can be as effective as SM-164 to prevent and/or eliminate metastatic breast cancer.

In conclusion, SM-164 is a promising agent to treat breast cancer metastases to bone and lung in the early stages of this process. However, the dosing regimen administered in this present study did not eliminate advanced bone metastases, despite slightly reducing their progression. Further studies will be required to determine if different dosing regimens or approaches more successfully eliminate advanced metastatic breast cancer.

## Materials and Methods

### Animals

Female athymic nude mice and C57Bl6 mice were purchased from Jackson Lab. C57Bl6 mice used for experiments were bred in house. All experimental protocols were approved by the University of Rochester Committee for Animal Resources. All methods were carried out in accordance with the American Veterinary Medical Association (AVMA) guidelines and regulations.

### Assay of Osteoclastogenesis in vitro

The effects of IAP antagonists on osteoclast (OC) formation were examined using our previously reported procedures^39,61^. Briefly, bone marrow (BM) was flushed from the tibiae and femora of C57Bl6 mice using α-MEM containing 2% FBS. The cells were incubated in NH_4_Cl solution for 15 min at room temperature to lyse red blood cells. 4 × 10^4^ cells were seeded in 96-well-plates with 5 ng/ml M-CSF for 2 d followed by treatment with 10 ng/ml RANKL (R&D system Cat# 462-TEC) +/− different compounds for 2-4 d when mature OCs typically are observed under inverted microscopy. The cells were then fixed with 10% neutral, phosphate-buffered formalin for 10 min and stained for tartrate-resistant acid phosphatase (TRAP) activity. TRAP^+^ cells with 3 or more nuclei were considered to be mature OCs.

### Assay of Osteoblast differentiation *in vitro*

Bone-derived mesenchymal stem cells (BdMPCs) from C57Bl6 mice^62^ or human MSCs (Lonza America, Alpharetta, GA, USA) were used to evaluate the effects of SM-164 on osteoblast (OB) differentiation. Briefly, the cells were seeded on 48-well-plates, 0.5 × 10^4^ cells per well, and cultured with 10% FBS in α-MEM at 37 °C in 5% CO_2_. From the second day, the cells were stimulated for OB differentiation with 25 μg/ml L-ascorbic acid and 5 mM β-glycerophosphate +/− SM-164 and TGFβ1 (R&D system Cat# 240-B). After 5–7 days, the cells were stained for alkaline phosphatase (ALP) activity using the ALP substrate, 1-Step BCIP/NBT assay (Thermo Scientific, Cat # 34042).

### Evaluation of breast cancer metastasis

MDA-MB-231^luci^ breast cancer cells (provided by Dr. Theresa Guise in the University of Texas Science Center San Antonio and now in Indiana University) expressing luciferase were generated by infecting the parental MDA-MB-231 cells with a CMV-Firefly luciferase lentivirus (Cellomics Technology, Halethorpe, MD) and inoculated into the left cardiac ventricle of 7-wk-old female athymic nude mice. On the 2^nd^ day when the inoculated cancer cells were expected to colonize in bone and other organs^25^, the mice were randomly divided into 4 groups, 8 mice per group, and treated with: 1) vehicle; 2) 3 mg/kg SM-164 (APExBIO, Cat# A8815) twice a day; 3) standard chemotherapy treatment (SCT), including 3 mg/kg adriamycin (ADR, Selleckchem, Cat# S1208) in the morning followed by 10 mg/kg cytoxan (CYT, Selleckchem, Cat# S2057) in the afternoon and 0.1 mg/kg zoledronate (ZOL, Sigma Cat# SML0223) the following day, with the cycle repeated each week; and 4) BV6 (APExBIO, Cat# B4653), another bivalent IAP inhibitor^26^, 10 mg/kg twice a week, as illustrated in Fig. 1A. In a similar parallel experiment, treatment was started on day 15 after cancer cell inoculation when BC bone metastases were well established and identified with BLI using an *in vivo* imaging system: IVIS Spectrum (PerkinElmer imaging system), and illustrated in Fig. 3A,B. The mice were monitored by BLI weekly and euthanized on day 29 to evaluate metastases in bone and other organs, such as lung, histologically. Mice were excluded if the cancer cells had not been successfully injected into the left cardiac ventricle, determined at 2 weeks with BLI showing signal distribution in the chest, but not in the other tissues, such as legs. Briefly, H&E-stained sections of decalcified long bones^61,62^ and of lung and other internal organs were used to evaluate metastasis, as assessed by the percentage of mice and long bones and lungs with metastases. Tumor burden was evaluated by measuring the tumor area using an OsteoMeasure Image Analysis System (OsteoMetrics). TRAP-stained sections of lower limbs and vertebral bones were used to evaluate trabecular bone mass and osteoclast parameters, as we described previously^61,62^.

### Immunofluorescence

Paraffin-embedded sections (4 µm thick) of decalcified L3-5 vertebrae and of lungs were incubated with anti-mouse RANKL monoclonal antibody (Ab) (Santa Cruz, sc-377079) overnight at 4 °C followed by Alexa Fluor 488-conjugated HRP-labeled secondary Ab (Abcam, ab150081) for 2 h at room temperature and DAPI counterstaining. Three images from each section were taken using a Zeiss fluorescence microscope and a x40 objective lens and the average number of RANKL^+^ cells was calculated.

### Establishment of ADR- and SM-164-resistant MDA-MB-231 cells

Parental MDA-MB-231 cells were treated with 1 nM of ADR for 3 days and the culture medium was replaced with fresh medium without ADR. Culture was continued until the surviving cells grew to sub-confluence. The cells were passaged to new dishes and the above procedure was repeated once. The above cycle was repeated with increasing doses of ADR (3, 10, 30 and 100 nM) to establish ADR-resistant cells (ADR-R). Similarly, parental MDA-MB-231 cells were treated with 3 nM SM-164 + 1 ng/ml TNFα for 2 days, and cells surviving after this treatment were continued in culture until the cells grew to sub-confluence. These cells were then passaged to new dishes and the above procedure was repeated with increasing doses of SM-164 (10, 30 and 100 nM), to establish SM-164-resistant cells (SM-R). The growth curves of parental and drug-resistant MDA-MB-231 cells were tested by passaging sub-confluent cells from wells of 6-well plates to 60-mm and then to100-mm dishes, respectively, and counting cell numbers in each passage.

#### Apoptosis assay

MDA-MB-231 cells were treated with different doses of SM-164 +/− 1 ng/ml TNFα (R&D system Cat# 210-GMP) overnight. The cells were stained with anti-Annexin V Ab (eBioscience, REF# 11-8005, or BioLegend Cat# 640941) and propidium iodide (PI, BioLegend Cat# 421301) and subjected to FACS to analyze Annexin V^+^PI^+/−^ apoptotic cells. To evaluate the effects of macrophages on SM-164-induced cancer cell apoptosis, 1.5 × 10^4^ GFP^+^ MDA-MB-231 cells that we generated by infecting parental MDA-MB-231 cells with a GFP-lentivirus were seeded on 60-mm dishes, and after 3 hours, 5 × 10^6^ bone marrow cells freshly isolated from WT mice, described above in the OC culture, were added and cultured in the presence of M-CSF +/− 10 ng/ml IL-4 (R&D system Cat# 404-ML) for 2 days. The cells then were treated with 3 nM SM-164 +/− the TNF inhibitor, TNF receptor IgG:Fc (TNFR:Fc)^38,39^, for an additional 16 hours. Annexin V^+^PI^+/−^ apoptotic cells in the GFP^+^ population were analyzed by flow cytometry.

#### Western Blot analysis

BM macrophages from C57Bl6 mice treated with M-CSF^61,63^ and BdMPCs^62^ from C57Bl6 mice treated with different compounds were lysed with M-Per mammalian protein extraction reagent (Thermo Scientific, Cat# 78501) containing a protease inhibitor cocktail (Sigma). Lysates (10–20 μg) were loaded in 10% SDS-PAGE gels and transferred onto polyvinylidene difluoride membranes. Following blocking in 5% milk, membranes were incubated overnight at 4 °C with Abs to human/murine Pan cIAP1&cIAP2 (R&D system Cat# MAB3400), murine TRAF3 (Santa Cruz, Cat# sc-947), murine monoclonal β-actin (Santa Cruz, Cat# sc-47778), or human/murine monoclonal GAPDH (Santa Cruz, Cat# sc-32233). After washing, the membranes were incubated with horseradish peroxidase-linked secondary Ab (Bio-Rad). The membranes were exposed to ECL substrate and signals were analyzed using a Bio-Rad imaging system.

#### Statistics

Descriptive statistics were presented by means and standard deviations for continuous variables. When data distributions are skewed, median and interquartile range were used instead. In addition, frequencies were presented for categorical variables. Comparisons between two groups were analyzed using Student’s two-tailed unpaired *t* test and those among 3 or more groups using one-way analysis of variance followed by Dunnett’s post-hoc multiple comparisons. When data distributions were not normal, Krusal Wallas was used to compare medians instead. Non-parameter statistical analyses were used for comparisons of frequency of bone and lung metastasis in mice. All analyses were performed at a two tailed 0.05 significance level.

## Supplementary information

Supplementary information.

## Data Availability

Cells are available upon signing a material transfer agreement.
