# Supplementary material for: The IAP Antagonist SM-164 Eliminates Triple-Negative Breast Cancer Metastasis to Bone and Lung in Mice
Source: Sci Rep. 2020 Apr 24;10:7004. doi: 10.1038/s41598-020-64018-z (PMC7181667; doi:10.1038/s41598-020-64018-z)
Supplement: Supplementary file 1 — Supplementary information. [file 41598_2020_64018_MOESM1_ESM.pdf]

The IAP Antagonist SM-164 Eliminates Triple-Negative Breast Cancer Metastasis to Bone and Lung in Mice

Wei Lei[1,2], Rong Duan[1], Jinbo Li[1], Xin Liu[1], Alissa Huston[3], Brendan F. Boyce[1], Zhenqiang Yao[1] *

Affiliations: [1]Department of Pathology and Laboratory Medicine, and Center for Musculoskeletal Research, University of Rochester Medical Center, Rochester, NY 14642, USA; [2]Department of Medical Imaging, Henan University First Affiliated Hospital, 357 Ximen Street, Kaifeng, Henan 475001, P.R. China. [3]Department of Medicine, Hematology/Oncology, University of Rochester Medical Center, Rochester, NY 14642, USA.

Supplementary data

Supplementary Fig. 1.

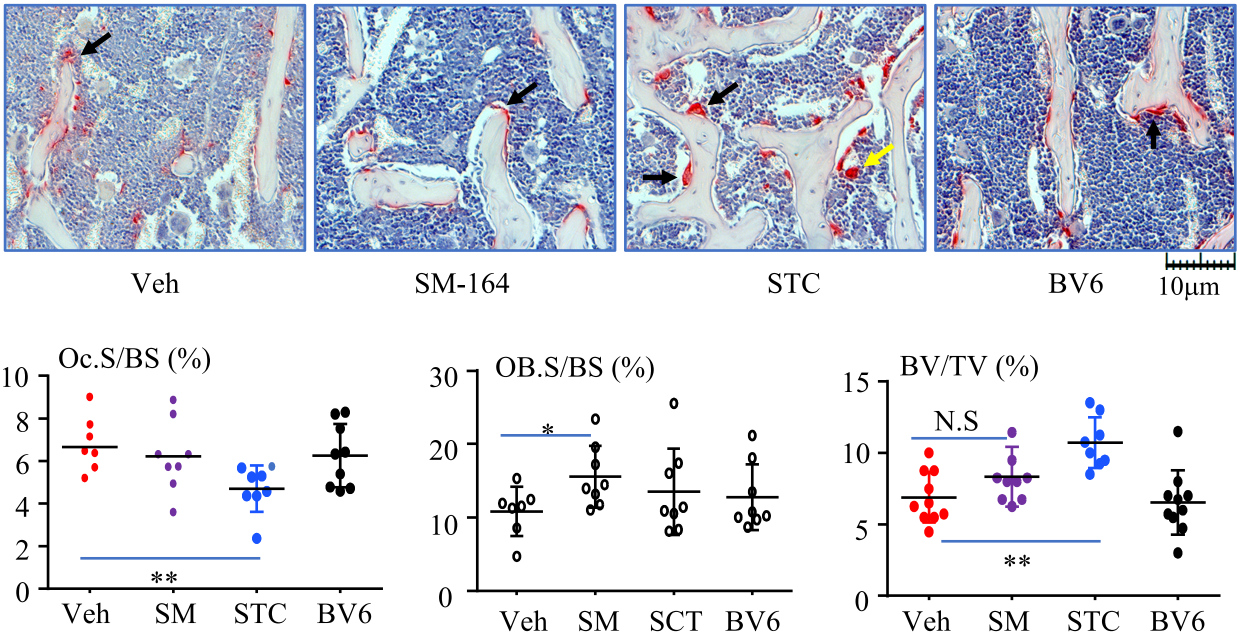

Supplementary Fig. 1. SM-164 does not affect bone turnover or bone mass in vertebrae without metastases. TRAP-stained sections of vertebrae without metastases, as in Figs. 1&2, were used to evaluate OC and OB surfaces and trabecular BV/TV in the metaphyseal bone adjacent to the end plates. Arrows indicate osteoclasts on bone surfaces. Osteoclasts are larger in the STC-treated mice than in the other groups and some are detached from the bone surface and have a rounded appearance (yellow arrow), typical of apoptotic osteoclasts. Bone volume values are higher in the SCT-treated mice, reflecting the inhibitory effects of zoledronate on osteoclast activity and survival. Veh (vehicle); SM (SM-164); SCT (standard chemotherapy).

Supplementary Figure 2

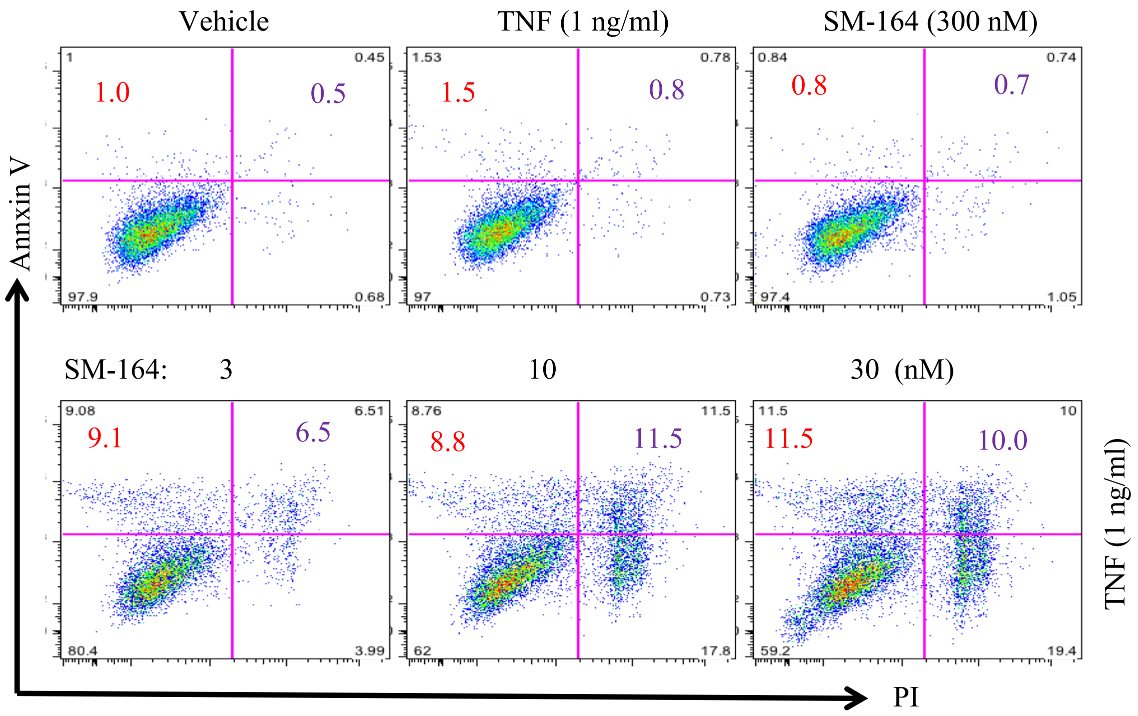

Supplementary Fig. 2. TNF triggers SM-164-induced MCF7 cell apoptosis. MCF7 breast cancer cells were treated with the indicated doses of SM-164 +/- 1 ng/ml of TNF overnight. Cells were stained with Annexin V and PI to analyze Annexin $V^{+}PI^{+/-}$apoptotic cells by flow cytometry. The experiments were repeated at least twice with similar results.

Supplementary Figure 3

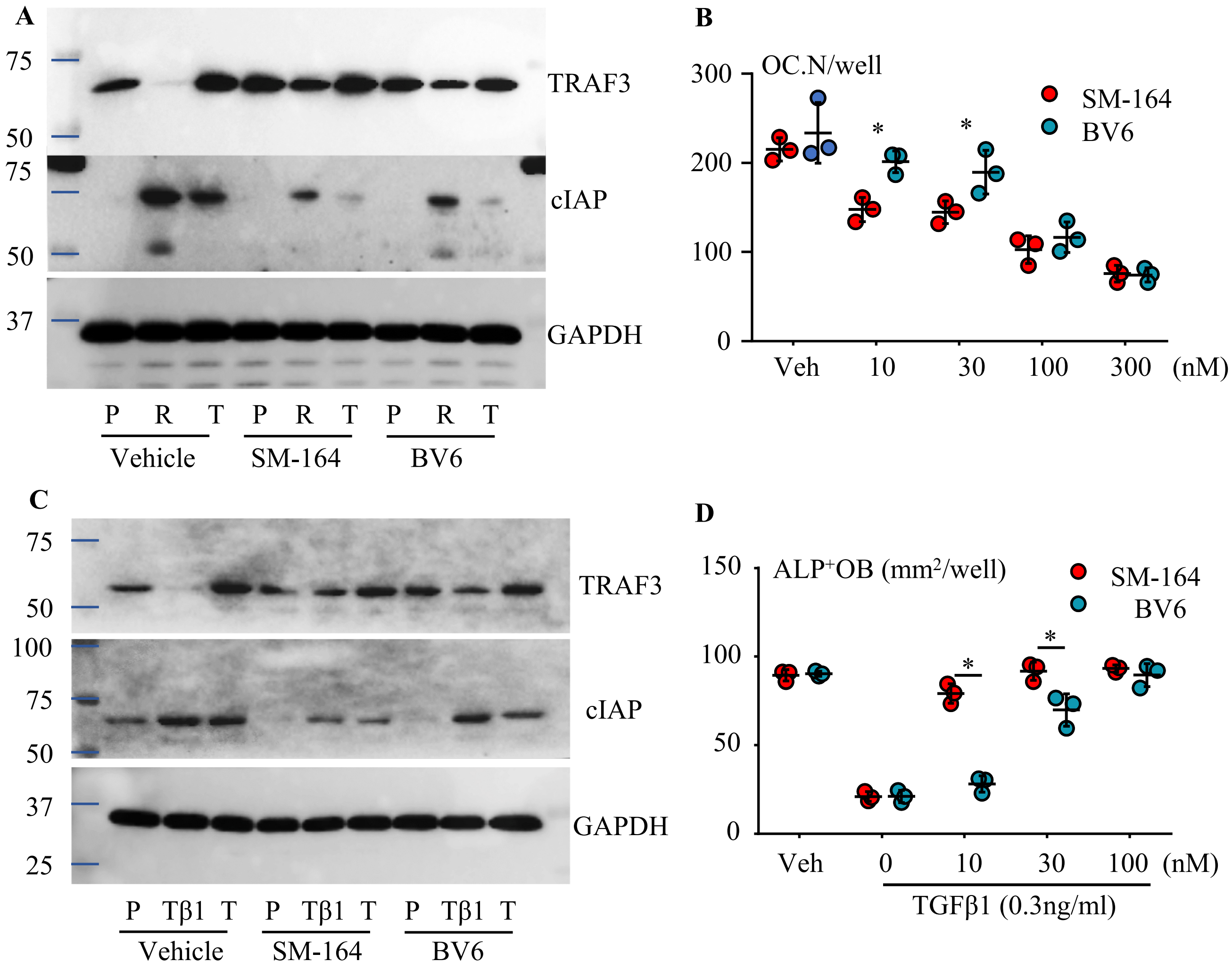

Supplementary Fig. 3. SM-164 more effectively inhibits OC and stimulates OB differentiation than BV6 by preventing TRAF3 degradation. (A) WT mouse BM cells were cultured with M-CSF to generate macrophages followed by treatment with PBS (P), RANKL (R) or TNF (T) plus vehicle, SM-164 or BV6 10 nM for 8 hrs. cIAP1/2 and TRAF3 protein levels were assessed by WB. (B) WT mouse BM cells were cultured with M-CSF and 10 ng/ml RANKL plus the indicated doses of SM-164 or BV6 for 4 days. TRAP^{+} OC numbers were counted. * $p<0.05$ vs. same dose of BV6. (C) WT mouse BdMSCs were treated with 1ng/ml TGFβ1 (Tβ1), or 3 ng/ml TNFα (T), +/- SM-164 (10 nM). Cell lysates were subjected to WB analysis of TRAF3, cIAP and β-actin. (D) BdMSCs from WT mice were induced for OB differentiation in the presence of the indicated doses of SM-164 or BV6 for 7 d. ALP^{+} OB area was evaluated. * $p<0.05$ vs. same dose of BV6.

Supplementary Fig. 4.

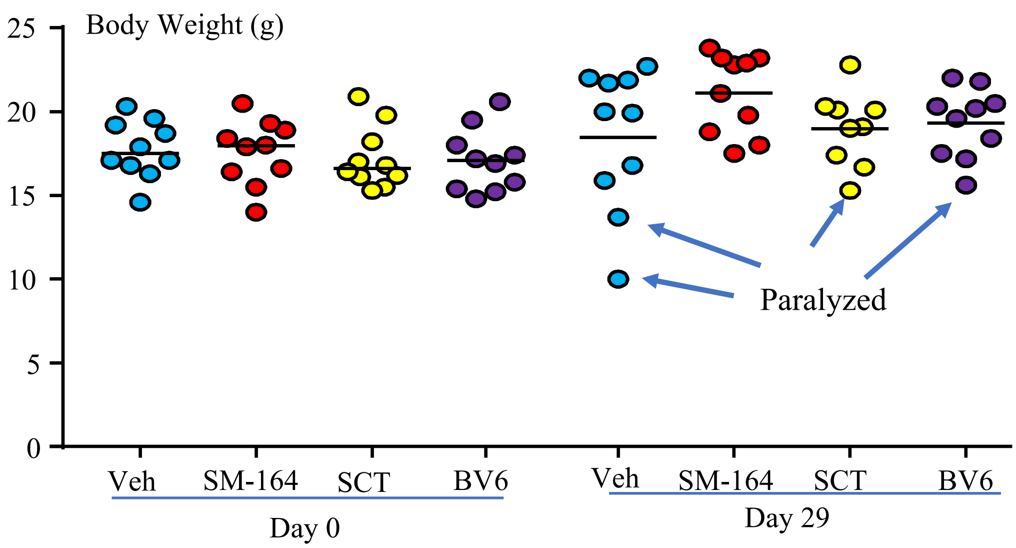

Supplementary Fig. 4. Effect of SM-164, standard chemotherapy (SCT) and BV6 on body weight of mice with metastases. Mice were weighed at the beginning and end of the experiment as Fig.1 &2. Arrows indicate mice that were paralyzed at time of euthanasia.

Supplementary Figure 5

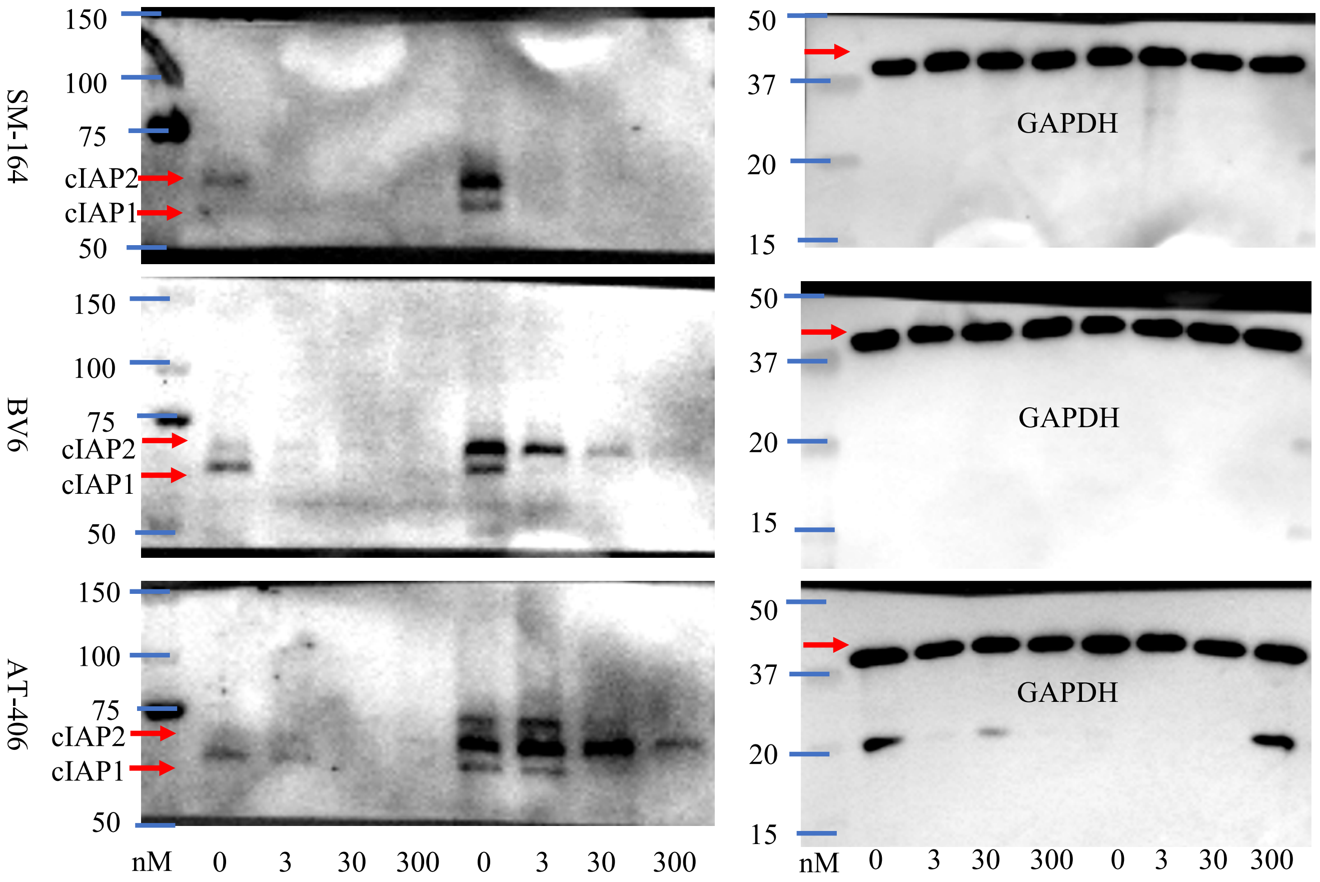

Full length gels of images presented in Fig. 4B

Supplementary Figure 6

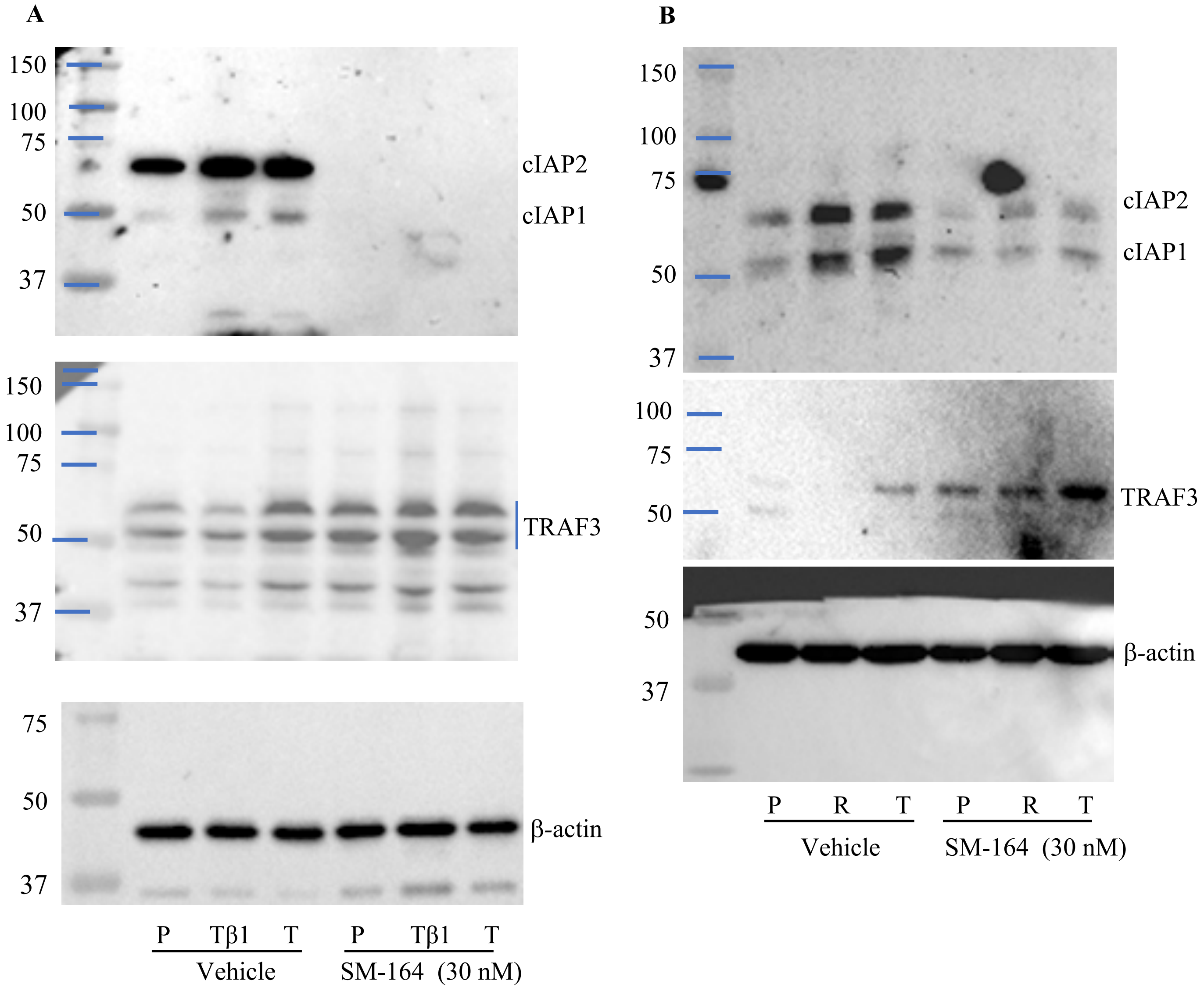

Full length gels of images presented in Fig. 5C (A) and Fig .5D (B). Tβ1 (TGFβ1) 1ng/ml; T (TNFα) 1 ng/ml.
